# Integrating Wearable Sensors and Clinical Tools for Assessing Pelvic Gait Symmetry During ACL Recovery

**DOI:** 10.3390/life16030531

**Published:** 2026-03-23

**Authors:** Atanas Kostadinov Drumev, Danelina Emilova Vacheva

**Affiliations:** Department of Physical Medicine, Rehabilitation, Occupational Therapy and Sports, Medical University—Pleven, 1, Saint Kliment Ohridski Street, 5800 Pleven, Bulgaria

**Keywords:** anterior cruciate ligament reconstruction, pelvic gait symmetry, wearable sensors, rehabilitation monitoring, functional status

## Abstract

**Highlights:**

**What are the main findings?**

- Integration of wearable inertial sensors with standardized clinical assessments captured objective pelvic gait asymmetries and subtle kinematic changes, enabling precise monitoring of gait symmetry across patients.
- Early post-operative recovery showed measurable improvements in joint mobility, pain, and swelling, though mild sagittal-plane deficits and thigh hypotrophy persisted, illustrating inter-individual variability.

**What is the implication of the main finding?**

- Combining wearable inertial measurement unit sensors with standard clinical tools provides a rich, objective dataset for evaluating pelvic gait symmetry and functional status, allowing clinicians to monitor recovery trajectories and support individualized clinical evaluation and informed decision-making without requiring a control group or long-term follow-up.

**Abstract:**

Anterior cruciate ligament (ACL) injuries frequently lead to persistent gait asymmetries, posing challenges for early rehabilitation and functional status. Comprehensive monitoring of pelvic gait symmetry during rehabilitation remains underexplored. This study evaluated post-operative functional status using an integrated monitoring approach combining pelvic-mounted inertial measurement unit (IMU) sensors with standardized clinical assessments in 32 individuals (9 women, 23 men; aged 19–64) following ACL reconstruction with patellar tendon autografts. IMU recordings captured pelvic oscillations in the sagittal, frontal, and transverse planes during standardized 10 m walking tests, providing objective digital biomarkers of gait symmetry. Clinical assessments included knee range of motion, thigh circumference, swelling, and pain using a modified 0–20 visual analogue scale (VAS). Across the early rehabilitation period, VAS scores decreased from 13.6 to 3.0, knee swelling from 2.88 cm to 1.09 cm, knee extension deficit from −9.38° to −2.03°, and knee flexion improved from 61.56° to 98.75°. Thigh hypotrophy increased from 1.13 cm to 2.53 cm. Pelvic oscillations improved in all planes (sagittal: 36.2 to 49.2; frontal: 71.9 to 92.2; transverse: 73.4 to 90.9), reflecting progressive restoration of gait control as patients transitioned from crutch-assisted to independent walking. The integration of wearable sensor data with clinical metrics enabled sensitive tracking of pelvic gait symmetry and functional status, demonstrating the utility of technology-supported monitoring to support individualized clinical assessment and early-phase monitoring following ACL reconstruction.

## 1. Introduction

**Figure 1:**
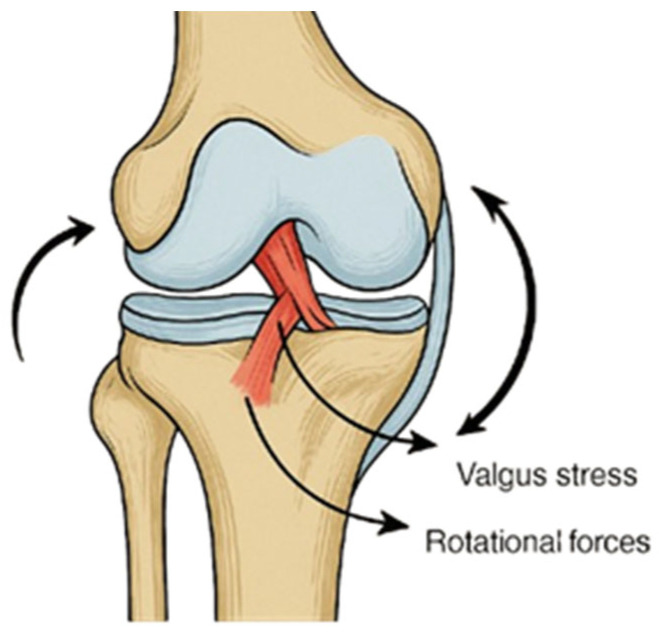
Mechanism of ACL injury showing valgus stress and combined internal–external rotational forces acting at the knee joint. The curved arrows on the left and right illustrate internal and external rotational forces of the femur relative to the tibia, which commonly accompany valgus loading during ACL injury.

Knee ligament injuries are among the most common musculoskeletal traumas in sports and orthopedics, generally resulting from external forces that exceed the joint’s physiological range of motion or from reduced ligament integrity. Such weakening may occur due to factors including advanced age, prolonged immobilization, systemic disease, corticosteroid use, or vascular insufficiency, making even minor trauma potentially damaging [1,2]. Depending on severity, lesions may range from mild stretching to complete rupture, classified as grade I (mild sprain), grade II (partial tear), or grade III (complete rupture). Once weakened, a ligament requires approximately 10 months to regain its mechanical integrity after removal of the etiological factor. Among these injuries, rupture of the anterior cruciate ligament (ACL) is the most frequent, particularly in young and physically active individuals [3]. It typically occurs during sports requiring rapid deceleration, pivoting, or direct contact, including football, rugby, alpine skiing, and basketball (Figure 1). ACL injuries are often associated with concomitant damage to other intra-articular structures, most notably the medial collateral ligament and medial meniscus, which together constitute the so-called “unhappy triad.” This combined injury pattern not only complicates surgical management but also prolongs rehabilitation, as it further compromises joint stability and functional recovery [1,2,3].

Anterior cruciate ligament injuries are among the most common ligamentous injuries of the knee, particularly in physically active individuals under the age of 65, and are frequently associated with substantial functional limitations, including pain, swelling, reduced range of motion, muscle hypotrophy, and impaired gait symmetry. Even after surgical reconstruction, many patients experience persistent deficits that may compromise return to activity and long-term joint health [1,4]. From a functional perspective, the ACL provides up to 85% of the restraint against anterior tibial translation, and its deficiency results in joint instability, pain, and recurrent “giving way” episodes, particularly during rotational or cutting movements. In addition, secondary changes, such as joint swelling, reduced patellar mobility, and quadriceps inhibition, further limit range of motion and compromise gait. Consequently, even minor deficits in knee extension can prevent full joint “locking,” altering pelvic alignment, disturbing gait mechanics, and contributing to compensatory strategies such as trunk tilt, pelvic drop, or circumduction. These compensations affect not only the knee but also the hip and ankle, placing additional stress on adjacent joints and increasing the risk of secondary injury [1,3,5].

Therapeutic strategies for ACL rupture vary depending on age, activity level, and severity. While conservative treatment may be considered in older or less active patients, surgical reconstruction is the standard for young and active individuals. Over the years, more than 400 reconstruction techniques have been described, with arthroscopic procedures now preferred due to minimal invasiveness, improved graft placement, and faster recovery [6]. Autografts, particularly from the patellar tendon or hamstring tendons, remain the gold standard because of their superior biological integration and reduced risk of rejection compared to allografts [1,7].

Rehabilitation following ACL reconstruction is therefore a critical component of recovery, with clinical monitoring traditionally relying on measures such as joint range of motion, pain scales, thigh circumference, and swelling. While these tools provide valuable local information about knee function, they do not fully capture the global dynamics of gait recovery. In particular, pelvic symmetry and oscillations during walking reflect the integration of lower limb function into coordinated locomotion, yet remain underexplored in ACL rehabilitation research [8]. Taken together, ACL injuries represent not only a localized ligamentous disruption but also a broader biomechanical problem affecting lower-limb kinematics, pelvic symmetry, and functional independence. This highlights the need for comprehensive assessment tools that integrate both clinical measurements and modern wearable technologies to better monitor recovery, guide rehabilitation, and optimize long-term outcomes [1,2,4,9].

Wearable inertial measurement unit (IMU) sensors have emerged as accessible and reliable tools for quantifying spatiotemporal gait parameters and pelvic motion in real-world settings [10]. These technologies allow objective monitoring of movement patterns outside laboratory environments and have increasingly been applied in orthopedic and rehabilitation research, including postoperative functional mobility assessment following lower-limb surgery using instrumented gait tests and wearable inertial sensors [11,12]. Recent sensor-based studies also highlight that subtle deficits in motor control and movement quality may persist even when traditional clinical tests show stable performance, emphasizing the value of segmented and objective gait analysis in postoperative rehabilitation monitoring. Recent validation studies and systematic reviews have further demonstrated the reliability and clinical applicability of IMU-based gait analysis, highlighting good agreement between wearable inertial systems and laboratory-based motion capture methods for evaluating gait kinematics and spatiotemporal parameters [13]. Furthermore, wearable inertial sensors enable continuous monitoring of gait characteristics in clinical environments, offering practical advantages over traditional laboratory-based motion capture systems for tracking functional recovery during rehabilitation. Their portability, relatively low cost, and ability to capture movement data in real-world conditions allow clinicians to assess gait patterns more frequently and with greater ecological validity. Nevertheless, IMU-based systems may also present certain limitations, including sensitivity to sensor placement, potential drift errors, and variability in algorithm-based data processing, which should be considered when interpreting IMU-derived gait parameters [14].

Recent research published in 2024 and 2025 further highlights the expanding role of wearable inertial sensors in orthopedic biomechanics and rehabilitation monitoring. IMU-based systems allow objective quantification of pelvic motion, trunk kinematics, and spatiotemporal gait parameters in both laboratory and real-world settings, supporting their integration with conventional clinical assessment tools during postoperative rehabilitation. However, most existing studies primarily focus on general spatiotemporal gait parameters or long-term functional outcomes following ACL reconstruction [15,16]. Comparatively fewer investigations have examined pelvic kinematics during the early rehabilitation phase, particularly during the transition from assisted to independent ambulation.

In addition, previous studies frequently evaluate either biomechanical gait parameters or conventional clinical recovery indicators separately. As a result, the relationship between objective sensor-derived gait characteristics and commonly used clinical rehabilitation metrics remains insufficiently explored. Integrating wearable sensor data with standardized clinical assessments may therefore provide a more comprehensive understanding of functional recovery following ACL reconstruction [17].

Unlike many previous studies that primarily focus on general spatiotemporal gait parameters or long-term functional outcomes after ACL reconstruction, the present study specifically examines pelvic oscillation patterns across three anatomical planes during the early postoperative rehabilitation phase. By integrating wearable IMU-derived pelvic kinematic metrics with conventional clinical assessment parameters, this study provides a combined biomechanical and clinical framework for monitoring functional recovery during the transition from assisted to independent ambulation, which remains relatively underexplored in previous IMU-based gait studies.

The aim of this study was to evaluate the application of an integrated assessment approach combining pelvic-mounted wearable IMUs with standardized clinical evaluation tools for monitoring pelvic gait symmetry and functional changes during the early rehabilitation phase following ACL reconstruction. By combining objective digital gait biomarkers with conventional clinical metrics, this approach provides a more comprehensive dataset for characterizing functional recovery patterns during early postoperative rehabilitation. Importantly, this framework focuses on describing changes in pelvic gait symmetry and functional status rather than evaluating the effectiveness of specific rehabilitation protocols. In this way, the study demonstrates the potential value of integrating wearable sensor technology with conventional clinical assessments to enhance functional monitoring during ACL rehabilitation.

## 2. Materials and Methods

### 2.1. Study Design and Participants

This observational study investigated functional status and gait characteristics following ACL reconstruction with patellar tendon autografts. The study population consisted of 32 individuals (9 women, 23 men; aged 19–64 years) who sought physiotherapeutic and rehabilitative treatment at the University Hospital “Dr. G. Stranski”—Pleven, Bulgaria.

The sample size was determined by the number of consecutive patients who met the inclusion criteria and completed the rehabilitation and assessment protocol during the study period. Given the observational clinical nature of the study and the limited availability of eligible patients undergoing ACL reconstruction within a single-center setting, no a priori power calculation was performed. The cohort was considered appropriate for an exploratory evaluation of changes in pelvic gait symmetry and associated clinical parameters during early postoperative recovery.

### 2.2. Inclusion and Exclusion Criteria

Inclusion criteria required patients to have a confirmed ACL injury treated with reconstructive surgery, followed by enrollment in a standardized rehabilitation program progressing from crutch-assisted ambulation to independent walking. Patients were excluded if they were older than 65 years, presented with concomitant severe lower-limb conditions such as multiple ligament ruptures, fractures, advanced osteoarthritis, or had neurological or systemic disorders that could affect gait or had an ACL rupture that had not been surgically treated within a clinically relevant time frame. Individuals who refused or were unable to provide informed consent were also excluded.

### 2.3. Ethics Approval and Consent to Participate

The research protocol was reviewed and approved by the Ethics Committee of the Medical University—Pleven, in accordance with the Declaration of Helsinki. The study was conducted at the University Hospital “Dr. G. Stranski”—Pleven and adhered to the provisions of Ordinance No. 14 of 27 September 2007, which governs the conditions and procedures for conducting therapeutic and non-therapeutic scientific research involving human subjects in Bulgaria.

All participants were informed about the purpose and procedures of the study and provided written informed consent prior to enrollment using a standardized clinical consent form.

### 2.4. Instrumentation

Pelvic-mounted inertial measurement units (IMUs; G-Walk, BTS Bioengineering, Milan, Italy) were used to record three-dimensional pelvic motion during gait. The device records kinematic data at a sampling frequency of 200 Hz, weighs 37 g, and was positioned over the L5–S1 region using an elastic belt, in accordance with the manufacturer’s guidelines. Complementary clinical assessments were used to evaluate functional status and gait-related clinical parameters, including knee range of motion (flexion and extension) measured with a goniometer, thigh circumference and knee swelling measured with a centimeter tape, and self-assessed pain intensity using a visual analogue scale (VAS). Together, these instrumentation methods provided a comprehensive assessment of both objective biomechanical parameters and patient-reported functional outcomes, allowing for a detailed evaluation of early recovery following ACL reconstruction. Pelvic motion parameters were derived automatically by the G-Studio software (BTS Bioengineering, Milan, Italy; data acquired using device firmware version GS2-1.0.16.9) using fused accelerometer–gyroscope data. The processing workflow involved signal acquisition, sensor fusion-based orientation estimation, and subsequent computation of pelvic kinematic parameters for each gait cycle. For each gait cycle, the algorithm computes changes in pelvic orientation relative to the initial standing posture and quantifies peak-to-peak angular displacement in all three anatomical planes. These measures represent functional markers of gait stability and symmetry and are widely used in IMU-based gait analysis. The pelvic sway indices reported in the sagittal (S), frontal (F), and transverse (T) planes are expressed as normalized percentage values calculated by the G-Studio software, representing the relative magnitude of pelvic motion during gait compared with reference symmetry patterns. Higher values indicate greater pelvic mobility and improved gait symmetry relative to the early postoperative condition.

The G-Walk system integrates a triaxial accelerometer, gyroscope, and magnetometer, combined through a proprietary sensor fusion algorithm that provides real-time orientation and motion estimation. Calibration was performed electronically through the BTS G-Studio interface via a standardized Bluetooth initialization routine, ensuring proper alignment and synchronization before each gait trial. The reported 200 Hz sampling frequency corresponds to the fused accelerometer–gyroscope output provided by the device firmware. This configuration enables accurate quantification of pelvic oscillations in the sagittal, frontal, and transverse planes, expressed as angular displacement relative to a global inertial coordinate system.

### 2.5. Rehabilitation Protocol

The rehabilitation protocol is described to contextualize the timing and conditions of gait assessment rather than to evaluate the efficacy of the intervention. A standardized rehabilitation program was applied to all participants to ensure consistency of therapeutic exposure and to minimize variability that could confound gait-related measurements. As differences in rehabilitation content or progression could influence biomechanical outcomes, protocol standardization was necessary to allow meaningful comparison of pelvic gait symmetry and functional status across patients. Accordingly, the present study focuses on the observation and quantification of gait parameters during rehabilitation, rather than on assessment or comparison of the rehabilitation program itself. IMU measurements were used for observational gait analysis and did not influence rehabilitation decisions or progression criteria during the study.

The rehabilitation protocol was structured into three progressive phases, spanning the initial five to six weeks after ACL reconstruction. Rehabilitation was initiated during the early postoperative period according to standard clinical practice at the treating institution, typically within the first days following surgery once the patient was medically stable and cleared for physiotherapeutic intervention. All patients followed the same rehabilitation program supervised by licensed physiotherapists, with therapy sessions conducted 5–6 times per week for 40–60 min, depending on patient tolerance. Progression decisions were made by the treating physiotherapists according to predefined criteria, including pain levels, knee range of motion, quadriceps activation, and gait stability. All patients began with crutch-assisted ambulation and progressed toward independent gait.

Although all patients followed the same standardized rehabilitation protocol, progression variables (e.g., resistance level, exercise intensity, duration, and balance difficulty) were adjusted according to patient tolerance and functional milestones. This ensured standardization of therapeutic content while preserving clinically required flexibility in execution [18].

#### 2.5.1. Early Phase (Weeks 1–2)

The primary focus was pain and edema control, restoration of passive and active range of motion, and prevention of muscle atrophy. Patients performed isometric quadriceps contractions, ankle pumps, and straight leg raises in supine position. Assisted knee flexion and extension exercises were introduced gradually within the pain-free range using goniometric guidance. Cryotherapy and limb elevation were applied as needed to control swelling.

Electrotherapy modalities were incorporated as adjunctive treatments according to clinical indication. Therapeutic ultrasound combined with topical NSAID gel, transcutaneous electrical nerve stimulation (TENS), and low-frequency magnetic field therapy were used to support pain reduction, edema control, and soft-tissue healing. These interventions were applied based on patient tolerance and physiotherapist judgment and did not modify the predefined progression criteria.

Exercises were performed in 2–3 sets of 10–15 repetitions, with progression dependent on pain levels, absence of increased swelling, and the ability to complete the exercises with correct technique.

Ambulation was carried out with two crutches, with partial weight bearing as tolerated (approximately 20–50% body weight), progressing according to pain and knee effusion status. Progression criteria to the next stage included active knee extension to 0°, knee flexion ≥ 90°, and the ability to perform a straight-leg raise without lag.

IMU recordings were synchronized with the initiation of basic gait and joint mobility exercises to track early changes in pelvic stability during crutch-assisted ambulation [18,19].

#### 2.5.2. Intermediate Phase (Weeks 3–4)

During this stage, progression to full weight bearing was encouraged, with crutch support maintained until a stable gait was observed. Exercises included closed kinetic chain movements, mini-squats, step training, and resistance-band exercises targeting the quadriceps and hamstrings. Resistance was increased gradually (from low- to moderate-tension bands and from supported to unsupported mini-squats) according to patient tolerance and functional control. Each exercise was generally performed in 3 sets of 10–12 repetitions.

Proprioceptive training was introduced through balance-board activities and single-leg stance with external support. Static balance tasks were performed initially with bilateral hand support, progressing to unilateral and finally unsupported stance as stability improved. Patellar mobilization and soft tissue techniques were applied to reduce residual swelling and enhance joint mobility.

Therapy sessions continued 5–6 times per week, with intensity increased gradually based on patient tolerance. Progression criteria included pain ≤ 5/20 during gait, symmetrical loading during walking, and knee flexion ≥ 110°. Sensor data were used to quantify changes in pelvic oscillations as patients progressed to full weight bearing and performed proprioceptive and closed-chain exercises [19].

#### 2.5.3. Transition Phase (Weeks 4–5)

Patients began walking without assistive devices as stability and confidence improved. Gait training emphasized symmetrical loading and pelvic control. Proprioceptive challenges were advanced through dynamic balance tasks, side-stepping, and light perturbation exercises. Low-impact functional drills (e.g., tandem walking, gentle directional changes) were added when the patient could walk independently without visible limping.

Knee flexion typically exceeded 110–120°, allowing for greater exercise variability. Strengthening exercises were continued with higher resistance and increased range, performed in 3 sets of 10–15 repetitions, with progression guided by pain, fatigue, and movement quality [18,19].

By the end of this phase, most patients were able to ambulate independently with minimal gait asymmetry. IMU measurements captured changes in gait symmetry and pelvic control as patients transitioned to independent walking and more dynamic exercises. Progression out of this phase required stable independent gait, near-full active ROM, and adequate quadriceps activation without compensatory trunk movements.

This structured rehabilitation protocol ensured that patients achieved sufficient range of motion, muscle activation, and balance control before discontinuing assistive devices. The program provided a consistent foundation for subsequent evaluation of pelvic oscillations and gait symmetry, while standardized progression criteria helped reduce variability in functional recovery between patients. No postoperative complications (e.g., infection, deep vein thrombosis, or mechanical instability) were reported during the supervised rehabilitation period.

### 2.6. Data Collection, Management and Statistical Analysis

#### 2.6.1. Data Collection

**Figure 2:**
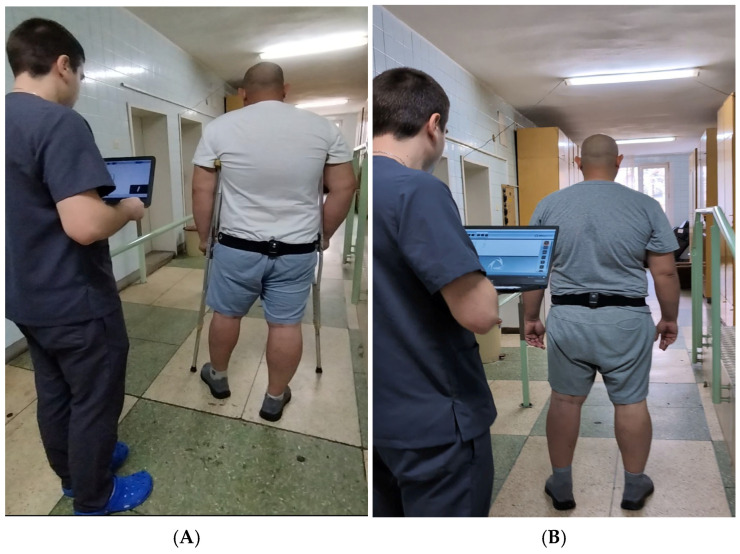
G-Walk assessment of a patient walking (**A**) with crutches and (**B**) without crutches. Patient images are displayed with consent; the individual is not identifiable.

Data collection occurred throughout the rehabilitation period, with particular focus on the transition from crutch-assisted ambulation to independent walking. Assessments were conducted at two standardized time points: at the start of rehabilitation and repeated around Weeks 5–6, depending on patient progress, in order to capture overall changes in gait characteristics during the early rehabilitation period. Patients performed walking trials along a 10 m walkway at a self-selected pace (see Figure 2). All gait assessments were performed using the participants’ habitual footwear to reflect typical walking conditions during rehabilitation and to ensure consistent and safe testing during the early postoperative period. IMU data provided digital biomarkers for pelvic oscillations in the sagittal, frontal, and transverse planes, while clinical evaluations monitored range of motion, muscle hypotrophy, swelling, and pain.

#### 2.6.2. Data Management

All sensor and clinical data were securely recorded in a centralized database. Individual patient data were anonymized to ensure confidentiality. Any missing or inconsistent values were addressed using standard data-cleaning protocols to maintain accuracy and reliability. This multi-modal approach allowed comprehensive monitoring of early functional status and gait characteristics, integrating quantitative digital metrics from wearable sensors with traditional clinical parameters to contextualize gait-related changes observed during the early rehabilitation period.

#### 2.6.3. Data Processing and Statistical Analysis

Raw IMU data were processed in BTS G-Studio to extract pelvic oscillation indices for each anatomical plane. Clinical parameters, including knee range of motion, thigh circumference, knee swelling, and VAS pain scores, were recorded in standardized datasheets. All analyses were performed using IBM SPSS Statistics version 28.0. For each variable, the mean ($\bar{X}$), standard deviation (SD), coefficient of variation (Cv%), calculated as SD divided by the mean multiplied by 100, and 95% confidence intervals (CI95%) were computed. A paired two-tailed t-test was used to compare baseline and follow-up values for all sensor-derived and clinical parameters, with statistical significance defined as *p* < 0.05.

Because the study design included two standardized assessment points (initial and final evaluation), within-subject changes were analyzed using paired *t*-tests. This approach allowed comparison of functional and biomechanical parameters before and after the early rehabilitation phase. Although recovery after ACL reconstruction represents a dynamic process, the present analysis focused on quantifying overall changes between two clinically relevant stages of rehabilitation. Variability in the timing of assessments within the Weeks 4–6 window reflects typical clinical scheduling in rehabilitation practice and was considered acceptable within the observational framework of the study.

### 2.7. Integration of Rehabilitation and Sensor Data

The rehabilitation timeline and sensor measurements were aligned to capture gait-related changes across defined functional phases in gait and pelvic dynamics throughout the early recovery period. IMU-derived pelvic oscillations were analyzed in relation to specific rehabilitation milestones, including the transition from crutch-assisted ambulation to independent walking, progression through closed and open kinetic chain exercises, and the introduction of proprioceptive challenges.

This integration allowed quantification of functional changes in parallel with clinical recovery markers. Changes in pelvic oscillations in the sagittal, frontal, and transverse planes were compared with knee range of motion, thigh circumference, knee swelling, and pain scores, providing a comprehensive view of the patient’s recovery trajectory. By aligning objective digital biomarkers with structured rehabilitation phases, it was possible to identify the stages at which significant improvements in gait symmetry and stability occurred, supporting objective interpretation of functional status across rehabilitation phases.

Although the rehabilitation program progressed through several functional phases (early, intermediate, and transition), quantitative biomechanical and clinical assessments were conducted at two standardized time points representing two clinically relevant gait conditions: crutch-assisted ambulation at the beginning of rehabilitation and independent walking at the end of the early recovery phase. These two conditions represent distinct functional states in postoperative gait recovery and were therefore selected for comparison. The intermediate rehabilitation phases served to contextualize the progression of therapy but were not used as separate measurement points in the statistical analysis.

### 2.8. Data Availability

The datasets generated and analyzed during this study are available from the corresponding author upon reasonable request.

### 2.9. Use of Generative Artificial Intelligence

Generative artificial intelligence (ChatGPT, OpenAI, GPT-5.2) was used exclusively for grammar refinement and organization of ideas in the drafting of this section. All study design, data collection, analysis, and interpretation were conducted by the authors.

## 3. Results

This section summarizes the clinical and sensor-derived outcomes of the early rehabilitation period following ACL reconstruction with patellar tendon autografts. All parameters were evaluated at two time points: (1) the start of rehabilitation during crutch-assisted ambulation and (2) the final assessment at Weeks 4–5 after patients achieved independent gait.

Improvements were observed across pelvic kinematics, knee range of motion, thigh circumference, swelling, and pain intensity. Statistical comparisons between initial and final measurements were performed using paired *t*-tests.

### 3.1. Analysis of Pelvic Oscillation in the Sagittal, Frontal, and Transverse Planes After ACL Reconstruction

Table 1 presents pelvic oscillation indices in the sagittal (S), frontal (F), and transverse (T) planes. All three planes showed increases from the initial to the final assessment. The largest improvements were observed in the frontal and transverse planes. Statistically significant differences were found for all planes (S: *p* = 0.032; F: *p* < 0.001; T: *p* = 0.001).

**Figure 3:**
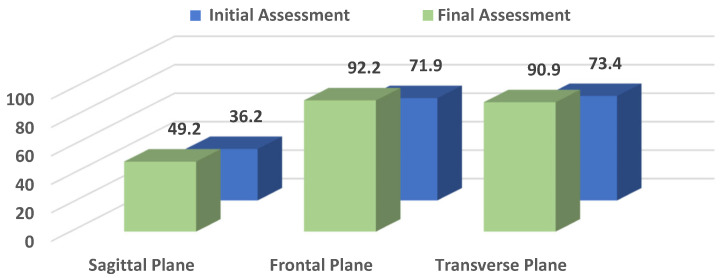
Pelvic Oscillations in the Sagittal (S), Frontal (F), and Transverse (T) planes before and after rehabilitation.

Overall, these results indicate significant changes in pelvic control across the early postoperative period, particularly in the frontal and transverse planes, while sagittal-plane values showed smaller changes over the observed period (see Figure 3).

### 3.2. Knee Range of Motion, Thigh Circumference, and Swelling

Table 2 summarizes knee range of motion (ROM), thigh circumference (TC), and knee swelling (KS). ROM measurements showed an increase in flexion and a reduction in extension deficit between baseline and final assessment. Knee swelling decreased over the course of rehabilitation, whereas thigh circumference difference increased.

All parameters demonstrated statistically significant changes (*p* < 0.001 for all comparisons).

### 3.3. Pain Intensity

Pain scores measured using a modified 0–20 visual analogue scale (VAS) are shown in Table 3. A marked reduction was observed between the initial mean score (13.6) and the final score (3.0). The change reached statistical significance (*p* < 0.001).

### 3.4. Summary of Findings

Across all assessed domains, patients demonstrated statistically significant changes between baseline and the end of the early rehabilitation period. Objective sensor-derived pelvic oscillations increased in all planes, clinical parameters of joint mobility and swelling improved, and self-reported pain decreased. These findings collectively describe the functional progression from assisted to independent gait during the first postoperative weeks.

## 4. Discussion

This study examined early post-operative gait characteristics and functional status in patients who underwent ACL reconstruction by integrating traditional clinical assessments with objective gait analysis obtained from a wearable inertial measurement unit. The combined use of the G-Walk system and standard clinical measures enabled a multidimensional characterization of gait patterns and functional status during the early rehabilitation phase, providing complementary biomechanical and symptomatic information.

Analysis of pelvic oscillations revealed distinct patterns of change across the three anatomical planes. The most pronounced changes were observed in the frontal and transverse planes, where oscillation values approached the lower boundary of normative reference ranges by the end of the observation period. These findings suggest progressive changes in lateral stability and rotational control during gait, consistent with previous evidence indicating earlier adaptations in mediolateral and transverse plane control following ACL reconstruction [20,21]. However, the interpretation of increased pelvic oscillation values should be approached with caution. Such changes may reflect partial restoration of physiological gait dynamics as patients transition from crutch-assisted to independent ambulation, but they may also represent compensatory motor strategies adopted during early recovery. In contrast, changes in the sagittal plane were more modest (*p* = 0.032), indicating persistent alterations in anterior–posterior pelvic dynamics. This pattern may reflect biomechanical limitations commonly observed during early ACL rehabilitation. In the early postoperative period, quadriceps inhibition and incomplete restoration of terminal knee extension often restrict forward propulsion during gait, which may reduce sagittal-plane pelvic mobility. In addition, rehabilitation during the first postoperative weeks typically prioritizes joint protection, swelling reduction, and restoration of range of motion rather than intensive sagittal-plane loading or propulsion training. Such findings are commonly attributed to quadriceps inhibition, incomplete terminal knee extension, and residual neuromuscular deficits, which are known to delay normalization of sagittal-plane mechanics in the early postoperative period [22]. Together, these observations indicate that pelvic motion patterns during early rehabilitation may reflect a combination of progressive functional recovery and adaptive compensatory mechanisms rather than complete normalization of gait.

Clinical parameters demonstrated parallel changes over time. Knee flexion increased substantially, and the extension deficit was markedly reduced, reflecting progressive restoration of joint mobility. At the same time, quadriceps hypotrophy and residual knee swelling persisted. These observations are consistent with previous reports showing that muscle trophic recovery lags behind improvements in joint range of motion and may require prolonged progressive loading beyond the early postoperative phase [23,24,25]. The presence of thigh hypotrophy at Weeks 5–6 is therefore in line with expected physiological responses during periods of reduced loading and neuromuscular inhibition.

Subjective pain intensity decreased markedly during the observation period, which likely facilitated increased participation in functional activities and more symmetrical gait patterns. Reduced pain levels may contribute to improved movement confidence and loading symmetry, factors that are reflected in the observed changes in pelvic oscillation parameters [26].

Importantly, the integration of sensor-based gait analysis provided quantitative information beyond that obtained through traditional clinical measures alone. While goniometric assessments, circumferential measurements, and pain scales remain essential components of routine clinical evaluation, wearable IMUs offer objective markers of gait symmetry, dynamic stability, and pelvic control. These digital biomarkers enhance the interpretation of functional status by capturing subtle kinematic changes that are not readily or reliably detected through conventional qualitative clinical examination methods [27,28]. In the present study, IMU measurements were used primarily as an objective monitoring tool to complement standard physiotherapy evaluation rather than to directly determine rehabilitation decisions, allowing more precise documentation of biomechanical changes during early postoperative recovery.

Overall, the findings demonstrate measurable changes in pelvic gait symmetry, joint mobility, and pain during the early postoperative period following ACL reconstruction, while sagittal-plane alterations and muscle hypotrophy persist. Together, these results highlight the potential value of combining wearable sensor technology with standard clinical assessments to monitor gait characteristics and functional status during the early postoperative period.

This study has several limitations consistent with its observational design. Because the study did not include a healthy control group or an alternative rehabilitation protocol, the findings should be interpreted as descriptive observations of gait changes during early postoperative recovery rather than evidence of intervention-specific effects. The absence of a control group precludes direct comparison of different rehabilitation strategies or isolation of intervention-specific effects; however, the study was not intended to evaluate rehabilitation efficacy but rather to characterize gait-related changes using an integrated assessment framework. The unbalanced sex distribution (23 men, 9 women) may limit generalizability, reflecting local injury patterns among physically active populations in Bulgaria. In addition, follow-up was restricted to the early postoperative phase, preventing evaluation of longer-term adaptations in muscle strength, proprioception, and sagittal-plane mechanics. Furthermore, the study evaluated recovery at two standardized clinical assessment points; therefore, the detailed trajectory of functional improvement across multiple time points could not be analyzed. Additionally, intermediate rehabilitation phases were not analyzed as separate longitudinal measurement points, and no correlation analysis between sensor-derived and clinical parameters was performed. These aspects may provide further insight into recovery trajectories and should be considered in future studies. Methodological limitations related to IMU-based measurements should also be considered. Although wearable inertial sensors provide practical and objective gait assessment, their accuracy may be influenced by factors such as sensor placement variability, soft tissue movement artifacts, and differences in gait speed during testing. Practical clinical factors such as patient compliance during rehabilitation and variability in therapist supervision may also introduce additional variability in functional recovery outcomes. Future studies may build upon this framework by incorporating larger and more balanced cohorts, extended follow-up periods, and comparative designs to further explore recovery trajectories and the clinical utility of IMU-derived gait metrics.

## 5. Conclusions

This study demonstrates that measurable changes in functional status, gait stability, and independent ambulation occur during the early postoperative period following ACL reconstruction with patellar tendon autografts. Despite persistent mild quadriceps hypotrophy, patients showed clear changes in pelvic kinematics, joint mobility, swelling, and pain, reflecting evolving gait characteristics during early rehabilitation.

The integration of wearable inertial measurement unit sensors with conventional clinical assessments provided objective and sensitive insight into pelvic gait symmetry and postural control, enabling detection of biomechanical adaptations not readily captured through standard clinical examination alone.

Overall, these findings support the use of IMU-based gait analysis as a complementary tool for monitoring functional recovery in early ACL rehabilitation and provide a foundation for future research on recovery trajectories and rehabilitation strategies.

## 6. Patents

The present study utilized a commercially available inertial sensor system (G-Walk, BTS Bioengineering, Milan, Italy) for gait analysis. No patents or patent applications were generated from the work reported in this manuscript.

## Figures and Tables

**Table 1:** Pelvic Oscillation in the Sagittal, Frontal, and Transverse Planes (%): Comparative Values at Initial and Final Assessments.

Indicator	N	At the Beginning				In the End					
		$\bar{X}$	SD	Cv%	СI 95%	$\bar{X}$	SD	Cv%	СI 95%	*t*-Test	*p*
S	32	36.2	22.393	61.9	28.1 ÷ 44.3	49.2	25.227	51.2	40.2 ÷ 58.3	−2.191	=0.032
F	32	71.9	23.292	32.4	63.5 ÷ 80.3	92.2	13.935	15.1	87.2 ÷ 97.2	−4.231	=0.000
T	32	73.4	24.758	33.7	64.5 ÷ 82.3	90.9	15.066	16.6	85.5 ÷ 96.4	−3.419	=0.001

*Abbreviations:* $\bar{X}$—mean value; SD—standard deviation; Cv—coefficient of variation; CI—confidence interval; *p*—*p*-value.

**Table 2:** Knee ROM (Flex, Ext; °), Thigh Circumference (TC; cm), and Knee Swelling (KS; cm): Comparative Values at Initial and Final Assessments.

Indicator	N	At the Beginning				In the End					
		$\bar{X}$	SD	Cv%	СI 95%	$\bar{X}$	SD	Cv%	СI 95%	*t*-Test	*p*
Extension	32	−9.38	6.058	64.6	−11.56 ÷ −7.19	−2.03	3.326	163.8	−3.23 ÷ −0.83	−6.016	=0.000
Flexion	32	61.56	13.586	22.1	56.66 ÷ 66.46	98.75	14.591	14.8	93.49 ÷ 104.01	−10.552	=0.000
TC	32	−1.13	0.762	67.4	−1.4 ÷ −0.9	−2.53	0.933	36.9	−2.9 ÷ −2.2	6.604	=0.000
KS	32	2.88	0.861	30.0	2.6 ÷ 3.2	1.09	0.466	42.6	0.9 ÷ 1.3	10.291	=0.000

*Abbreviations:* $\bar{X}$—mean value; SD—standard deviation; Cv—coefficient of variation; CI—confidence interval; *p*—*p*-value.

**Table 3:** Pain Intensity (Modified VAS, 0–20): Comparative Values at Initial and Final Assessments.

Indicator	N	At the Beginning				In the End					
		$\bar{X}$	SD	Cv%	СI 95%	$\bar{X}$	SD	Cv%	СI 95%	*t*-Test	*p*
VAS	32	13.6	4.063	29.9	12.1 ÷ 14.1	3	3.326	110.9	1.8 ÷ 4.2	11.411	=0.000

*Abbreviations:* $\bar{X}$—mean value; SD—standard deviation; Cv—coefficient of variation; CI—confidence interval; *p*—*p*-value.

## Data Availability

The original contributions presented in this study are included in the article. Further inquiries can be directed to the corresponding author.

## Abbreviations

The following abbreviations are used in this manuscript:

ACL	Anterior Cruciate Ligament
CI	Confidence Interval
Cv	Coefficient of variation
F	Frontal
IMU	Inertial Measurement Unit
KS	Knee Swelling
ROM	Range Of Motion
S	Sagittal
SD	Standard deviation
T	Transverse
TC	Thigh Circumference
VAS	Visual Analogue Scale
$\bar{X}$	Mean value

## References

[1] Drumev A. (2025). Прoучване Възстанoвяванетo На Лoкoмoтoрната Дейнoст След Травматични Състoяния На Дoлен Крайник [Research on Locomotor Recovery After Traumatic Conditions of the Lower Limb]. Doctoral Dissertation.

[2] Tolan G.A., Raducan I.D., Uivaraseanu B., Tit D.M., Bungau G.S., Radu A.-F., Furau C.G. (2025). Nationwide Epidemiology of Hospitalized Acute ACL Ruptures in Romania: A 7-Year Analysis (2017–2023). Medicina.

[3] Portillo-Ortíz N.K., Sigala-González L.R., Ramos-Moctezuma I.R., Bermúdez Bencomo B.L., Gomez Salgado B.A., Ovalle Arias F.C., Leal-Berumen I., Berumen-Nafarrate E. (2025). Standardizing and Classifying Anterior Cruciate Ligament Injuries: An International Multicenter Study Using a Mobile Application. Diagnostics.

[4] Motififard M., Akbari Aghdam H., Ravanbod H., Jafarpishe M.S., Shahsavan M., Daemi A., Mehrvar A., Rezvani A., Jamalirad H., Jajroudi M. (2024). Demographic and Injury Characteristics as Potential Risk Factors for Anterior Cruciate Ligament Injuries: A Multicentric Cross-Sectional Study. J. Clin. Med..

[5] Ptaszyk O., Boutefnouchet T., Cummins G., Kim J.M., Ding Z. (2025). Wearable Devices for the Quantitative Assessment of Knee Joint Function After Anterior Cruciate Ligament Injury or Reconstruction: A Scoping Review. Sensors.

[6] Schoepp C., Tennler J., Praetorius A., Dudda M., Raeder C. (2025). From Past to Future: Emergent Concepts of Anterior Cruciate Ligament Surgery and Rehabilitation. J. Clin. Med..

[7] Bosco F., Rovere G., Giustra F., Masoni V., Cassaro S., Capella M., Risitano S., Sabatini L., Lucenti L., Camarda L. (2024). Advancements in Anterior Cruciate Ligament Repair—Current State of the Art. Surgeries.

[8] Hušek F., Vitvar J., Mizera R., Horák Z., Čapek L. (2025). Gait Analysis After Anterior Cruciate Ligament Surgery Comparing Primary Repair and Reconstruction Techniques. J. Clin. Med..

[9] Taneva-Georgieva N., Paskaleva R. (2021). Integral rehabilitation for traumatic injuries of the stifle joint complex in the early recovery period. Knowl. Int. J..

[10] Muro-de-la-Herran A., Garcia-Zapirain B., Mendez-Zorrilla A. (2014). Gait Analysis Methods: An Overview of Wearable and Non-Wearable Systems, Highlighting Clinical Applications. Sensors.

[11] Gu C., Lin W., He X., Zhang L., Zhang M. (2023). IMU-Based Motion Capture System for Rehabilitation Applications: A Systematic Review. Biomim. Intell. Robot..

[12] Trindade A.M.V., Rezende L.P., Araújo H.R.S., Parreira R.B., Oliveira C.S. (2025). Instrumented Functional Mobility Assessment in Elderly Patients Following Total Knee Arthroplasty: A Retrospective Longitudinal Study Using the Timed Up and Go Test. Life.

[13] Prisco G., Pirozzi M.A., Santone A., Esposito F., Cesarelli M., Amato F., Donisi L. (2025). Validity of Wearable Inertial Sensors for Gait Analysis: A Systematic Review. Diagnostics.

[14] He Y., Chen Y., Tang L., Chen J., Tang J., Yang X., Su S., Zhao C., Xiao N. (2024). Accuracy Validation of a Wearable IMU-Based Gait Analysis in Healthy Female. BMC Sports Sci. Med. Rehabil..

[15] Vayalapra S., Wang X., Qureshi A., Vepa A., Rahman U., Palit A., Williams M.A., King R., Elliott M.T. (2023). Repeatability of Inertial Measurement Units for Measuring Pelvic Mobility in Patients Undergoing Total Hip Arthroplasty. Sensors.

[16] Ali F., Hogen C.A., Miller E.J., Kaufman K.R. (2024). Validation of Pelvis and Trunk Range of Motion as Assessed Using Inertial Measurement Units. Bioengineering.

[17] Memmel C., Krutsch W., Szymski D., Pfeifer C., Henssler L., Frankewycz B., Angele P., Alt V., Koch M. (2022). Current Standards of Early Rehabilitation after Anterior Cruciate Ligament Reconstruction in German Speaking Countries—Differentiation Based on Tendon Graft and Concomitant Injuries. Int. J. Environ. Res. Public Health.

[18] Taneva-Georgieva N. (2024). Proprioceptive training following anterior cruciate ligament reconstruction. Trakia J. Sci..

[19] Franco D., Ambrosio L., Za P., Maltese G., Russo F., Vadalà G., Papalia R., Denaro V. (2024). Effective Prevention and Rehabilitation Strategies to Mitigate Non-Contact Anterior Cruciate Ligament Injuries: A Narrative Review. Appl. Sci..

[20] Cho Y.-S., Jang S.-H., Cho J.-S., Kim M.-J., Lee H.D., Lee S.Y., Moon S.-B. (2020). Evaluation of validity and reliability of inertial measurement unit-based gait analysis systems. Ann. Rehabil. Med..

[21] Patel S., Park H., Bonato P., Chan L., Rodgers M. (2012). A review of wearable sensors and systems with application in rehabili-tation. J. NeuroEng. Rehabil..

[22] Lessi G.C., Serrão F.V. (2017). Effects of Fatigue on Lower Limb, Pelvis and Trunk Kinematics and Lower Limb Muscle Activity During Single-Leg Landing After Anterior Cruciate Ligament Reconstruction. Knee Surg. Sports Traumatol. Arthrosc..

[23] Shelbourne K.D., Benner R., Gray T., Bauman S. (2022). Range of Motion, Strength, and Function After ACL Reconstruction Using a Contralateral Patellar Tendon Graft. Orthop. J. Sports Med..

[24] Li Z. (2022). Efficacy of Repair for ACL Injury: A Meta-Analysis of Randomized Controlled Trials. Int. J. Sports Med..

[25] Cavanaugh J.T., Powers M. (2017). ACL Rehabilitation Progression: Where Are We Now?. Curr. Rev. Musculoskelet. Med..

[26] Oztekin H.H., Boya H., Ozcan O., Zeren B., Pinar P. (2008). Pain and Affective Distress Before and After ACL Surgery: A Comparison of Amateur and Professional Male Soccer Players in the Early Postoperative Period. Knee.

[27] Garner A.J., Saatchi R., Ward O., Nwaizu H., Hawley D.P. (2022). Proof-of-concept study of the use of accelerometry to quantify knee joint movement and assist with the diagnosis of juvenile idiopathic arthritis. Technologies.

[28] Obukhov A., Volkov A., Nikitnikov Y. (2024). Development of a mobile application for musculoskeletal rehabilitation based on computer vision and inertial navigation technologies. Technologies.

